# Draft genome sequence of the anatoxin-a producing cyanobacterium *Tychonema bourrellyi* B0820 isolated from the epilimnion of the deep Alpine Lake Garda

**DOI:** 10.1128/MRA.00844-23

**Published:** 2023-10-19

**Authors:** Nico Salmaso, Adriano Boscaini, Leonardo Cerasino, Massimo Pindo, Federica Pinto, Nicola Segata, Claudio Donati

**Affiliations:** 1 Research and Innovation Centre, Fondazione Edmund Mach, San Michele all'Adige, Italy; 2 NBFC, National Biodiversity Future Center, Palermo, Italy; 3 Department of Cellular, Computational and Integrative Biology, University of Trento, Trento, Italy; California State University, San Marcos, California, USA

**Keywords:** cyanobacteria, *Tychonema*, draft genome, full shotgun, anatoxin-a, lake, non-axenic culture

## Abstract

We report the draft genome sequence of strain B0820 of the cyanobacterium *Tychonema bourrellyi* isolated from the epilimnion of Lake Garda and assembled from a metagenome of a non-axenic culture. The strain analyzed was shown to produce anatoxin-a, a potent neurotoxin that can cause fatal intoxication in exposed organisms.

## ANNOUNCEMENT

Following the discovery (1) of anatoxin-a (ATX)-producing strains of *Tychonema bourrellyi* (2, 3) in the epilimnion of the southern Alpine lakes, other ATX-producing *Tychonema* species in freshwater mats were described in Germany, where they caused dog deaths (4, 5).

*Tychonema* strain B0820 was collected from the epilimnion of Lake Garda (45.69 N, 10.72 E) in August 2020. Under the microscope, a single filament was isolated, washed repeatedly with sterilized Z8 medium, and cultured in the same medium at 20°C with 25 µmol m^−2^ s^−1^ illumination (6). The 16S rRNA (OR088434) and ATX (OR124837) sequences [Sanger sequencing (6)] showed 100% identity with previously reported *T. bourrellyi* genes. Using standard LC-MS methods (7), strain B0820 was shown to produce anatoxin-a.

For metagenomic analysis, to reduce the bacteria in the non-axenic culture, filaments were diluted in 1 L of sterilized Z8 medium, filtered through sterilized 80 µm mesh, resuspended in 100 mL Z8 medium, and filtered through 5 µm cellulose nitrate membranes (Whatman). DNA was extracted using PowerWater Kit (Qiagen). The library was prepared using Kapa HyperPlus Kit (Roche) and processed by 150-bp paired-end sequencing on an Illumina NovaSeq 6000, which generated 98,364,812 paired-end reads. Unless otherwise stated, the software was run with default parameters. Reads were checked with FastQC 0.11.9 (8). Removal of residual adapters and trimming (trimq = 20 maq = 20 maxns = 0 minlen = 35, ftm = 5, tpe, tbo) were performed using bbduk 38.95 (https://jgi.doe.gov). After correction with metaSPAdes 3.15.3 (9) (--only-error-correction), the paired reads were assembled with megahit 1.2.9 (10) (--presets meta-sensitive), yielding 6,411 contigs larger than 1,000 bp, with a total size of 160 Mbp and N50 of 201,376 bp. The draft genomes were binned using CONCOCT 1.1.0 (11), MetaBAT 2 2.12.1 (12), and MaxBin 2 2.2.7 (13), and results combined using DAS Tool 1.1.6 (14). The *T. bourrellyi* bin was checked and confirmed with anvi’o 7.1 (15). The genome was 5.37 Mbp assembled into 223 contigs, with N50 39,298 bp, GC content 44.6%, and coverage 336×. Using the phylum Cyanobacteria and the order Oscillatoriales as marker lineages, CheckM 1.2.2 (16) estimated a completeness of 98.9% and 99.3%, respectively, with almost no evidence of contamination (0.07% and 0.08%) and no strain heterogeneity. The taxonomy of the draft genome was confirmed by GTDB-Tk 2.1.1 (17) and a phylogenomic analysis using PhyloPhlAn 3.0.3 (18), which indicated coincidence (ANI = 99.6) with a strain previously isolated from Lake Garda (19).

The genome annotation was completed by the NCBI Prokaryotic Genome Annotation Pipeline (PGAP 6.5) (20), which identified 5,125 coding sequences and 52 tRNAs. After blastn (nr/nt) analysis, the 16S rRNA and *rbcX* genes from the draft genome were 99.9%–100% and 100% identical, respectively, to previously reported *T. bourrellyi* genes. A polyketide synthase protein gene cluster associated with ATX biosynthesis was identified by blastn (99%-100% query coverage) with 93.1% and 93.4% identity to the anatoxin-a synthetase gene clusters of *Anabaena* sp. 37 (JF803645) and *Cuspidothrix issatschenkoi* (LT984882), respectively; the same sequence showed 99.8%–100% identity to *anaF* sequences previously determined in *T. bourrellyi* isolates (query coverage 3%–7%).

## Data Availability

This Whole Genome Shotgun project has been deposited at DDBJ/ENA/GenBank under the accession JAVCBQ000000000. The version described in this paper is version JAVCBQ000000000.1. The sequences are publicly available under BioProject accession number PRJNA979152, BioSample accession number SAMN35566361, and SRA accession number SRR25705839. Sanger sequences of 16S rRNA and anaF genes of strain B0820 were deposited to GenBank with accession numbers OR088434 and OR124837, respectively.
